# Human Intellectual Disability Genes Form Conserved Functional Modules in *Drosophila*

**DOI:** 10.1371/journal.pgen.1003911

**Published:** 2013-10-31

**Authors:** Merel A. W. Oortveld, Shivakumar Keerthikumar, Martin Oti, Bonnie Nijhof, Ana Clara Fernandes, Korinna Kochinke, Anna Castells-Nobau, Eva van Engelen, Thijs Ellenkamp, Lilian Eshuis, Anne Galy, Hans van Bokhoven, Bianca Habermann, Han G. Brunner, Christiane Zweier, Patrik Verstreken, Martijn A. Huynen, Annette Schenck

**Affiliations:** 1 Department of Human Genetics, Nijmegen Centre for Molecular Life Sciences, Donders Institute for Brain, Cognition and Behaviour, Radboud University Medical Centre, Nijmegen, The Netherlands; 2 CMBI, Nijmegen Centre for Molecular Life Sciences, Radboud University Medical Centre, Nijmegen, The Netherlands; 3 VIB, Center for the Biology of Disease, Leuven, Belgium; 4 KU Leuven, Center for Human Genetics & Leuven Institute for Neuroscience and Disease (LIND), Leuven, Belgium; 5 Department of Pathology, Radboud University Medical Centre, Nijmegen, The Netherlands; 6 Institut de la vision, Paris, France; 7 Max Planck Institute for Biology of Ageing, Cologne, Germany; 8 Institute of Human Genetics, Friedrich-Alexander-Universität Erlangen-Nürnberg, Erlangen, Germany; The Wellcome Trust Centre for Human Genetics, University of Oxford, United Kingdom

## Abstract

Intellectual Disability (ID) disorders, defined by an IQ below 70, are genetically and phenotypically highly heterogeneous. Identification of common molecular pathways underlying these disorders is crucial for understanding the molecular basis of cognition and for the development of therapeutic intervention strategies. To systematically establish their functional connectivity, we used transgenic RNAi to target 270 ID gene orthologs in the *Drosophila* eye. Assessment of neuronal function in behavioral and electrophysiological assays and multiparametric morphological analysis identified phenotypes associated with knockdown of 180 ID gene orthologs. Most of these genotype-phenotype associations were novel. For example, we uncovered 16 genes that are required for basal neurotransmission and have not previously been implicated in this process in any system or organism. ID gene orthologs with morphological eye phenotypes, in contrast to genes without phenotypes, are relatively highly expressed in the human nervous system and are enriched for neuronal functions, suggesting that eye phenotyping can distinguish different classes of ID genes. Indeed, grouping genes by *Drosophila* phenotype uncovered 26 connected functional modules. Novel links between ID genes successfully predicted that MYCN, PIGV and UPF3B regulate synapse development. *Drosophila* phenotype groups show, in addition to ID, significant phenotypic similarity also in humans, indicating that functional modules are conserved. The combined data indicate that ID disorders, despite their extreme genetic diversity, are caused by disruption of a limited number of highly connected functional modules.

## Author Summary

Intellectual Disability (ID) affects 2% of our population and is associated with many different disorders. Although more than 400 causative genes (‘ID genes’) have been identified, their function remains poorly understood and the degree to which these disorders share a common molecular basis is unknown. Here, we systematically characterized behavioral and morphological phenotypes associated with 270 conserved ID genes, using the *Drosophila* eye and photoreceptor neurons as a model. These and follow up approaches generated previously undescribed genotype-phenotype associations for the majority (180) of ID gene orthologs, and identified, among others, 16 novel regulators of basal neurotransmission. Importantly, groups of genes that show the same phenotype in *Drosophila* are highly enriched in known connectivity, also share increased phenotypic similarity in humans and successfully predicted novel gene functions. In total, we mapped 26 conserved functional modules that together comprise 100 ID gene orthologs. Our findings provide unbiased evidence for the long suspected but never experimentally demonstrated functional coherence among ID disorders. The identified conserved functional modules may aid to develop therapeutic strategies that target genetically heterogeneous ID patients with a common treatment.

## Introduction

Intellectual Disability (ID) is defined by an IQ below 70, deficits in adaptive behavior and an onset before the age of 18. ID disorders are among the most common and important unmet challenges in health care due to their tremendous phenotypic and genetic heterogeneity [1], [2]. Many ID disorders are monogenic, and disease gene identification over the past decade has been very successful. More than 400 causative genes (referred to as ID genes) have been identified, providing unique stepping stones for understanding the molecular basis of cognition in health and disease. Some ID genes appear to work together in specific pathways and processes, such as Rho GTPase pathways, MAP kinase signalling and synaptic plasticity [3], [4]. This has led to the suggestion that ID genes highlight key molecular networks that regulate human cognition [1], [2], [5]–[7]. Such networks are of wide interest for both fundamental neuroscience and translational medicine, and can pave the way for developing treatment strategies [2]. However, their identification is limited by the paucity of available information on the function of most ID genes. Model organisms such as the mouse have effectively been used as experimental systems to gain insights into ID gene function and neuropathology [8]. Because such studies are time and cost intensive, ID research, whether *in vitro* or *in vivo*, has so far not moved beyond studying individual or small groups of genes. Novel approaches are required to allow functional studies to catch up with disease gene identification. We used *Drosophila melanogaster* as the model organism for this study. Genes, pathways, and regulatory networks are well-conserved between flies and humans [9]. *Drosophila* provides numerous approaches to investigate defects in neuronal function and behavior. Furthermore, fly models of selected ID disorders have already provided major insights into ID pathologies and have triggered the first therapeutic approaches [10], [11]. The efficiency of this organism and its available genome-wide toolboxes [12], [13] make *Drosophila* a powerful model to generate comparative phenotype datasets that can provide global insights into ID gene function and connectivity.

Here, we present a large-scale *in vivo* assessment of ID gene function and an *in silico* analysis of their *Drosophila* phenotypes and phenotype classes. We investigated the role of 270 evolutionarily conserved ID gene orthologs (referred to from here on as ‘*Drosophila* ID genes’) in the *Drosophila* compound eye, a highly organized array of ommatidia and photoreceptor neurons that allows for simultaneous assessment of neuronal function and physiology, and for multiparametric morphological analysis.

This comparative survey revealed a large number of novel functions for *Drosophila* ID genes including previously unappreciated regulatory roles in basal neurotransmission. It identified novel phenogroups in *Drosophila* that show phenotypic coherence in humans and molecular modules that can predict novel gene functions. Our study demonstrates that ID disorders converge on a limited number of highly connected functional modules.

## Results

### A Large Scale Screen of ID Gene Function in the *Drosophila* Eye

**Figure 1:**
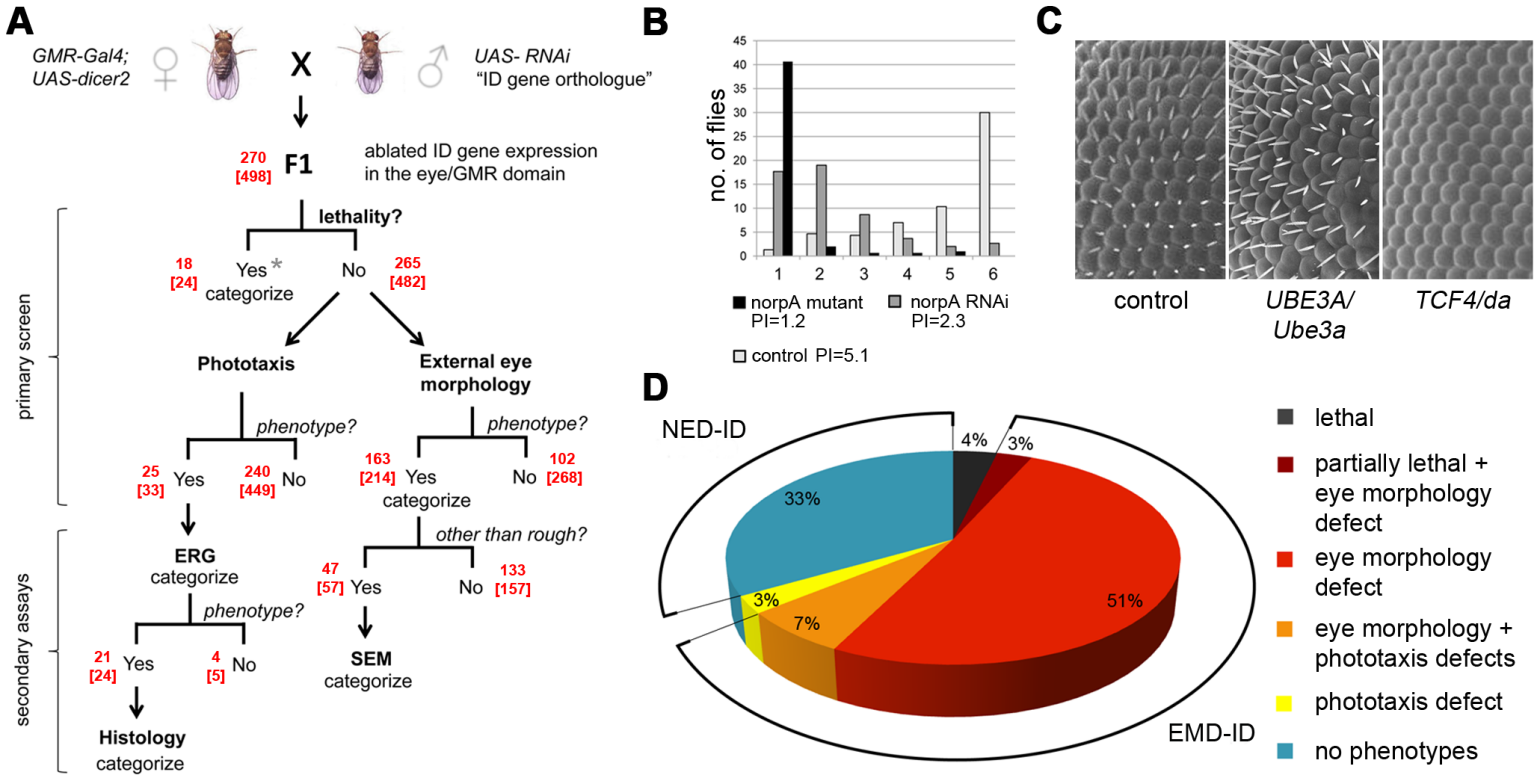
Large scale screen of Intellectual Disability genes in *Drosophila* and phenotype distribution. (A) Screening program. In the primary screen, lethality, phototaxis and external eye morphology were scored. The numbers of *Drosophila* ID genes and RNAi lines (in brackets) are added in red color at each step. Note that total numbers do not add up, as multiple phenotypes can be assigned to one gene. Secondary assays: Electroretinogram (ERG), Scanning electron microscopy (SEM), histology. Lethal genes (asterisk) were subjected to analysis of lethality upon pan-neuronal ablation. (B) Proof of principle for the phototaxis assay and RNAi approach, using a known blind mutant (*norpA*, in black), *norpA* RNAi (vdrc 21490, in dark grey) and a control (in light grey). Distribution of genotypes over the 6 phototaxis vials. PIs are indicated. The severity of phenotypes was *norpA*>*norpA* RNAi. The phototaxis device and further proof of principle data are shown in **Figure S1.** (C) Proof of principle for RNAi-based defects in external eye morphology. Knockdown of *Ube3a* and *da* results in the expected loss of bristles and rough eye phenotypes. (D) Distribution of 270 screened ID gene orthologs into phenotype classes. The three indicated classes with morphological defects form the group of eye morphology defective *Drosophila* ID (EMD-ID) genes. Genes without any phenotype define no eye defect *Drosophila* ID (NED-ID) genes. All RNAi genotypes and their associated phenotypes are provided in **Table S1A**.

To generate novel insights into the neuronal and molecular basis of cognitive (dys)function, we set out to manipulate established monogenic causes of ID in humans using *Drosophila* as a model. At the start of this project we conducted a systematic, manually curated disease gene survey. Of the identified 390 ID genes, 285 were conserved in *Drosophila* (for curation criteria and orthology see Materials and Methods). 95% of these genes, 270 *Drosophila* ID genes, can be targeted with *Drosophila* transgenic conditional RNA interference (RNAi) lines from an established validated toolbox [12], [14], [15]. This approach is a suitable approximation to the human disease conditions since (partial) loss of gene function is thought to be the causative mechanism for more than 250 of the 270 ID genes investigated (see Materials and Methods and **Table S1A**). We used a total of 498 RNAi lines, including two independent RNAi constructs per gene whenever available (**Table S1A**). To maximize the reliability in our primary screen, we selected lines which exceed previously determined quality criteria that guaranteed high reproducibility (see Materials and Methods, discussion, and Neumüller et al. [15]). Our strategy to ablate *Drosophila* ID gene expression primarily in the developing eye, including the photoreceptor neurons, was directed at identifying i) *Drosophila* ID genes that, if perturbed, cause defects in neuronal function, ii) *Drosophila* ID genes that affect viability, and iii) *Drosophila* ID genes that control different aspects of eye morphology (**Figure 1A**). We reasoned that these three classes and their subcategories might break down the large number of *Drosophila* ID genes into phenogroups, containing genes with a coherent function. Systematic targeting of a defined, larger group of genes in the eye and phenotypic characterization of various phenotypes has to our knowledge not previously been reported. Thus the degree to which phenotypes would be obtained was unknown.

The fast phototaxis assay is an efficient and robust test for neuronal function. It is based on the fly's innate behavior to move towards a light source [16], critically depends on proper performance of photoreceptor neurons, and can be quantified using the Phototaxis Index (PI) (**Figure S1A**). We optimized the assay using known vision mutants and their corresponding RNAi lines (**Figure S1B,C**). Under the chosen screening conditions (*GMR-Gal4; UAS-dicer2* driver line, 28°C) all proof of principle RNAi lines showed strong defects, phenocopying their mutant phenotypes (**Figure 1B**, **Figure S1B,C**), which validated the efficiency of our approach.

In parallel to phototaxis, *Drosophila* ID gene knockdown progeny were examined for morphological eye phenotypes. As proof of principle for this additional approach, we tested RNAi lines against two *Drosophila* ID genes with reported eye phenotypes: *ubiquitin protein ligase 3a* (*ube3a*), the *Drosophila* ortholog of *UBE3A* implicated in Angelman syndrome, and *daughterless* (*da*), the ortholog of *TCF4* implicated in Pitt-Hopkins syndrome. RNAi lines against both genes resulted in the expected defects, rough eyes [17] and complete loss of interommatidial bristles [18], respectively (**Figure 1C**). Progeny of the *GMR-Gal4; UAS-dicer2* driver crossed to the genetic background line of the RNAi lines served as controls in all experiments of our study. Controls showed no considerable eye phenotypes (see Materials and Methods) and wildtype-like performance in the phototaxis assay.

In our screen, RNAi against the majority of all *Drosophila* ID genes (180 genes, 67%) resulted in lethal, phototactic or morphologic phenotypes (**Figure 1D**, **Table S1B,C**). Knockdown of the remaining 90 *Drosophila* ID genes (33%) did not yield functional or morphological eye phenotypes. The identified phenotype groups are described below.

### Essential *Drosophila* ID Genes

Eighteen *Drosophila* ID genes (7%) gave rise to (partial) lethality and are thus essential in the targeted tissues (**Table S1B,C**). The eye driver *GMR-Gal4* has recently been reported to show some expression outside the eye, which likely accounts for the lethality that was already reported by others [12], [19], [20]. Expression of these 18 genes was subsequently knocked down specifically in neurons, using the pan-neuronal driver *elav-Gal4* (**Figure 1A**, grey asterisk). Only *ERCC2* (human gene symbol)/*Xpd* (*Drosophila* gene symbol) and *TPI*/*Tpi* did not show lethality when ablated in neurons. Sixteen of the 18 *GMR-Gal4*-induced lethal genes also showed 100% lethality before adult stages upon selective neuronal knockdown (**Table S1B**). Thus, 16 *Drosophila* ID genes that are essential in neurons were identified using this strategy.

### *Drosophila* ID Genes Required for Different Aspects of Basal Neurotransmission

**Figure 2:**
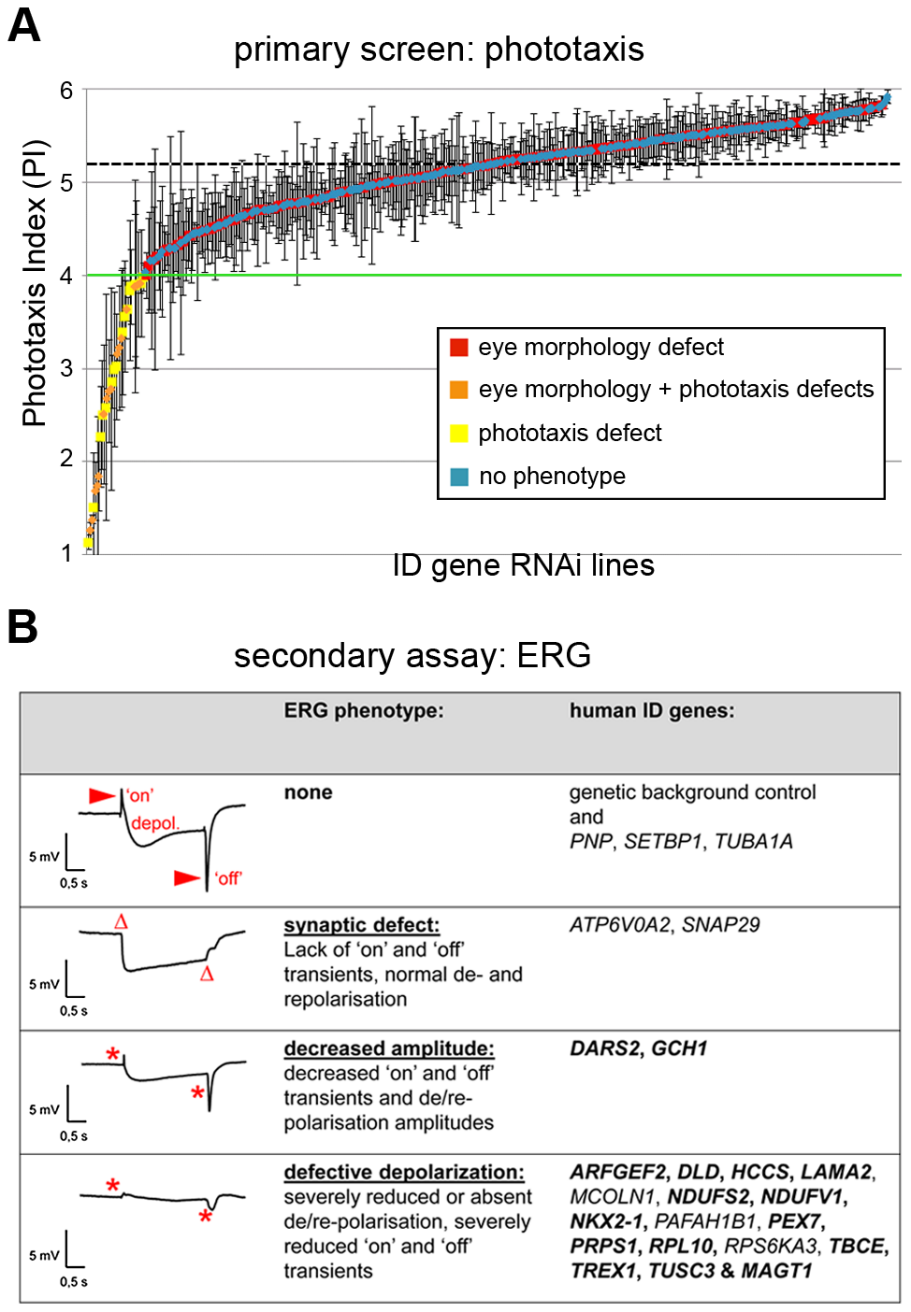
Phototaxis and electrophysiology defects of *Drosophila* ID models. (A) Results of phototaxis screen. Average Phototaxis Indexes (PIs) of all assayed RNAi lines. Error bars indicate Standard Deviations in triplicate experiments. Horizontal black dashed line indicates the average PI of the genetic background controls. Green line indicates the threshold defining a phototaxis defect. Note the random distribution of eye morphology defects (in orange and red) along the entire range of PIs. (B) Electroretinogram (ERG) phenotypes of phototaxis defective ID conditions. Three ERG defective categories can be distinguished. Per category, a representative profile and the human ID gene symbols are shown. Genes that have not previously been associated with basal neurotransmission defects are highlighted in bold. The novelty of these data is discussed in **Table S2**. Red arrowheads indicate the synaptic response (‘on’ and ‘off’ transients). Note the complete absence (Δ) or strong reduction of transients (*) in the mutant conditions. In the latter two categories, also receptor potentials (depolarization) are affected. Genotypes are provided in **Table S1B.**

Ablating ID gene orthologs in the *Drosophila* eye and quantitatively assessing phototaxis yielded PIs between 1.1 and 5.9. Using a stringent cut-off of <4.0 to define phototaxis defects, we identified 25 phototaxis defective *Drosophila* ID genes (**Figure 2A** **, Table S1B**). Among these is the ortholog of *ATP6V0A2*, the vacuolar proton pumping ATPase subunit *Vha100-1*, mutations in which have been previously identified in an unbiased large scale phototaxis screen [21].

Electroretinograms (ERGs) were performed as a secondary screen to confirm that defects in phototaxis behavior are indeed caused by defective photoreceptor function and to further dissect the cause of defective vision in these ID models. ERGs are extracellular field recordings that measure the potential difference between the photoreceptor layer and the remainder of the fly body during light stimulation, revealing photoreceptor receptor transients (de- and repolarization) and synaptic communication (‘on’ and ‘off’ transients) [22]. Of the 24 *Drosophila* ID genes tested, we confirmed that 21 exhibited defective neuronal physiology. Of these, *ATP6V0A2/Vha100-1* and *SNAP29/usnp* showed isolated synaptic defects characterized by normal receptor potentials but complete absence of ‘on’ and ‘off’ transients (**Figure 2B**). Two further *Drosophila* ID genes, *DARS2* and *GCH1*, exhibited decreased amplitudes of receptor transients and reduced synaptic signalling, whereas the majority (17 of 21) of phototaxis hits were characterized by nearly absent depolarization and only residual synaptic communication (**Figure 2B**). In summary, we identified 21 *Drosophila* ID genes that are required either specifically for synaptic transmission or more broadly for basal neurotransmission and physiology. Only *Vha100-1* has been previously demonstrated to be required for synaptic transmission in *Drosophila* photoreceptors. The majority of genes (16 of 21) had not been previously implicated in basal neurotransmission in any system or organism (**Figure 2B**, **Table S2**).

### Histological Analysis of ERG Defective *Drosophila* ID Conditions

**Figure 3:**
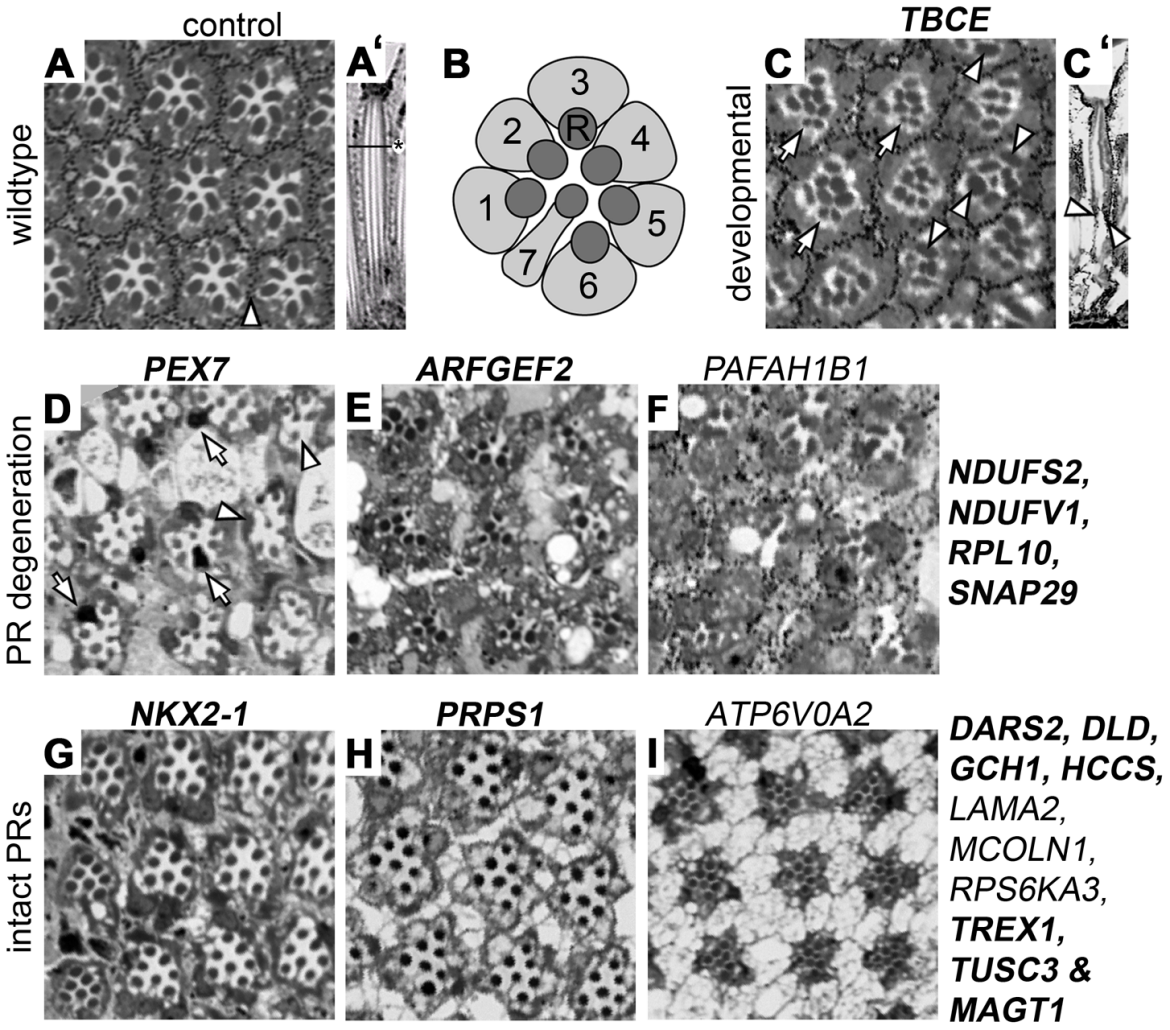
Histological analysis of *Drosophila* ID gene knockdown eyes with ERG defects. (A) Wildtype pattern of an ommatidia array in a transversal section of a control retina. Arrowhead: pigment cells (A′) Longitudinal section of a single ommatidium, lens to the top. The horizontal line and asterisk mark the level of the transversal section in all other panels. Dark structures (A, A′) are rhabdomeres, the photosensitive domains of Photoreceptors (PRs). (B) Schematic drawing of PR 1–7 in their typical stereotype pattern. PR cytoplasms in light grey. R: rhabdomeres. (C–I) A selection of histological sections of *Drosophila* ID gene knockdown eyes. Corresponding human gene names are indicated. Genes that have not previously been associated with histological phenotypes are highlighted in bold. The novelty of these data is discussed in **Table S2**. (C,C′) Transversal and longitudinal sections reveal a *TBCE* mutant phenotype of developmental origin. Arrowheads: bulky rhabdomeres, arrows: mis-positioned PR8s. (D–F) and genes indicated to their right: neurodegeneration in several ID conditions. Arrows in D point to black photoreceptor cytoplasms, arrowheads to single lost PRs/rhabdomeres. Massive loss of PRs can be seen in panels E and F. (G–I) and genes indicated to their right: structurally intact photoreceptors. Genotypes are provided in **Table S1B.**

Internal eye architecture and the state of photoreceptors were monitored in order to obtain further insights into the cellular basis of the identified neurophysiological defects. Each wild-type ommatidium contains eight photoreceptors, organized in a stereotypical pattern (**Figure 3A,B**). Histological sections of ERG-defective *Drosophila* ID conditions detected a number of phenotypes (**Figure 3** **, Table S1B**). For example, knockdown of TBCE/tbce, implicated in hypoparathyroidism-retardation-dysmorphism syndrome, showed structural defects of developmental origin. R8 photoreceptors, normally located underneath photoreceptor 7, failed to be maintained in their appropriate proximal position and thus appeared in distal sections (**Figure 3C**). Moreover, rhabdomere extension towards the retina base, a process taking place during pupal development, failed in the majority of ommatidia (**Figure 3C′**) leading to distally accumulated “bulky” rhabdomeres (**Figure 3C**). This defect has recently been associated with regulators of the actin cytoskeleton that are linked to ID [23], [24]. In contrast, RNAi against several ERG defective *Drosophila* ID genes, including *PEX7*, *ARFGEF2* and *PAFAH1B1* caused neuronal degeneration of variable degrees, identifying a role for the encoded proteins in neuronal maintenance (**Figure 3D–F**). Thirteen of 21 ERG defective *Drosophila* ID conditions, including *NKX2-1*, *PRPS1* and *ATP6V0A2* knockdown animals, showed intact and properly organized photoreceptors (**Figure 3G–I**). Some of these conditions showed darker photoreceptor cytoplasm or pigment cell abnormalities (**Figure 3G–I** and **Table S1B**).

In summary, we identified genes required for neuronal development or maintenance among the ID orthologs that cause neurophysiological defects. In 20% of these cases the data confirm or extend previous findings. In the majority of instances (80%) these functions are novel (**Figure 3**, **Table S2**).

### Eye Morphology Defects of *Drosophila* ID Genes

**Figure 4:**
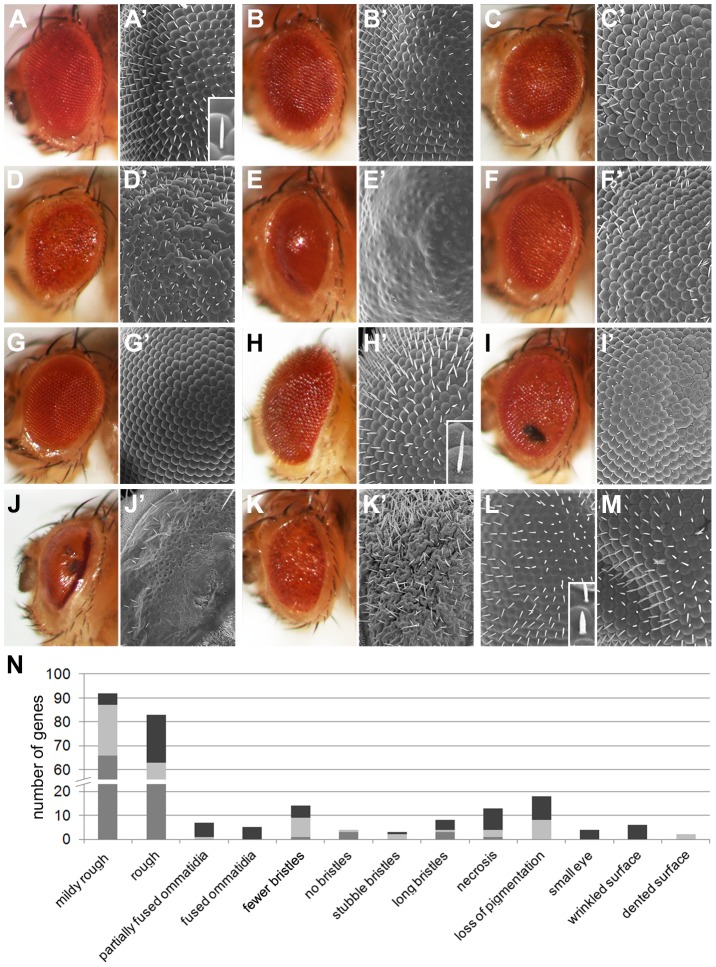
Eye morphology defects of *Drosophila* ID models. (A–M) Representative eye morphology defects in *Drosophila* ID gene knockdown eyes. (A,A′) Wild-type. (B,B′) PNP, mildly rough. (C,C′) ABCD1, rough. (D,D′) RAB39B, ommatidia partially fused, loss of pigmentation and wrinkled surface. (E,E′) MED12, fused ommatidia and loss of pigmentation. (F,F′) AFF2, fewer bristles and rough eyes. (G,G′) FGFR2/3, no bristles. (H,H′) TSC2, long bristles (compare inset H′ to inset A′). (I,I′) TBCE, mildly rough and necrosis. (J,J′) SURF1, loss of pigmentation, necrosis, small eye and fused ommatidia. (K,K′) DMD, small eye, rough, wrinkled surface, long bristles. (L′) ASL, stubble (-like) bristles and fused ommatidia. Bristles are short and thick (compare inset l′ with inset a′). (M′) HSD17B10, rough eye and dented surface. (N) Total number of *Drosophila* ID genes with the indicated morphologic eye phenotypes. Medium grey bars represent isolated eye phenotypes. Light grey bars represent phenotypes that co-occurred with mildly rough or rough phenotypes. In the case of mildly rough phenotype it indicates co-occurrence with rough, and vice versa. Dark grey bars represent phenotypes that co-occurred with eye phenotypes other than rough or mildly rough. Insets with single magnified bristles in A′, I′ and L′ correspond to a height of 35 mm. Genotypes are provided in **Table S1B.**

External eye morphology was systematically assessed in the primary screen to determine whether multiparametric phenotyping could identify which *Drosophila* ID genes work together in common developmental processes or molecular pathways. Thirteen phenotypic categories were identified: mildly rough, rough, partially fused ommatidia, fused ommatidia, fewer bristles, no bristles, stubble bristles, long bristles, necrosis, loss of pigmentation, small eye, wrinkled surface and dented surface (**Figure 4A–M** **and Table S1B**). 163 *Drosophila* ID genes showed at least one of these morphological phenotypes, which were classed as eye morphology defective. Mildly rough and rough phenotypes were the most numerous. Other defects occurred frequently in combination with these and/or with other phenotypes (**Figure 4N**). In all, RNAi-mediated knockdown of *Drosophila* ID genes in the eye generated a series of specific phenotype categories and identified a large number of genes with a role in the development of this tissue.

Interestingly, the frequency of morphological phenotypes among the phototaxis defective genes was very similar to their overall frequency in our screen. Thus, these phenotype classes do not significantly correlate (p = 0.13, hypergeometric test), which is also illustrated by the random distribution of morphologic phenotypes along the entire spectrum of phototactic performance (**Figure 2A**). We conclude that vision and external eye morphology do not depend on the same genetic/molecular machineries and provide a largely independent assessment of gene function.

### Eye Morphology Phenotypes Characterize Genes Associated with Nervous System Expression, Development and Function

We next sought to determine whether *Drosophila* eye morphology defects could provide insights into conserved functional networks that underlie human ID disorders. To our knowledge, such a correlation has not previously been evaluated. Therefore, we first examined the expression, annotated functions and protein interactions, comparing EMD (Eye Morphology Defective)- and NED (No Eye Defect)- ID genes (classes indicated in **Figure 1D**; the terms EMD- and NED-ID genes refer to *Drosophila* genes throughout the text).

Based on EST data from 45 human tissues [25], the human orthologs of both EMD-ID and NED-ID genes were widely expressed. For each gene we determined the tissue in which its normalized expression is highest (normalized for overall expression per tissue; see Materials and Methods). We found that the largest fraction among EMD-ID orthologs (9.8%, 16 genes) had their highest normalized expression in human ‘nerve’ tissue. This was also, among all tissues, the tissue where EMD- and NED-ID gene orthologs differ the most, as only 2.2% (2 genes) of NED-ID orthologs had their highest expression in ‘nerve’ (4.4 fold enrichment EMD-ID over NED-ID, P = 0.046). In contrast, the tissue in which most NED-ID orthologs had their highest expression was parathyroid (11.1%, 10 genes) (**Figure S2A**).

EMD-ID genes were also enriched for nervous system-related phenotypes in FlyBase, such as neuroanatomy, neurophysiology and photoreceptor defects (**Figure S2B**) as well as for Gene Ontology (GO) terms and KEGG pathways related to neuronal processes in humans. In contrast, NED-ID genes were enriched for GO terms related to metabolic processes (**Figure S2C,D**). The frequencies of human postsynaptic density proteins (hPSD; 1458 proteins, ∼7% of human genes [25]) among human orthologs of EMD- versus NED-ID proteins were also compared. In general hPSD proteins were significantly enriched among all ID genes (3 fold, χ^2^, P = 3.65e-18, ID genes (58) vs. human genome (1458)) but to a different extent among the two eye phenotype-based classes of ID genes: 25% of human orthologs of EMD-ID genes encoded hPSD proteins (3.4 fold enriched vs. genome, 41 proteins, **Table S3**), compared to 13% of human orthologs of NED-ID genes (1.8 fold enriched vs. genome, 12 proteins, **Table S3**). hPSD proteins are thus enriched by ∼2 fold among human orthologs of EMD-ID genes relative to NED-ID genes (χ^2^, P = 0.04).

**Figure 5:**
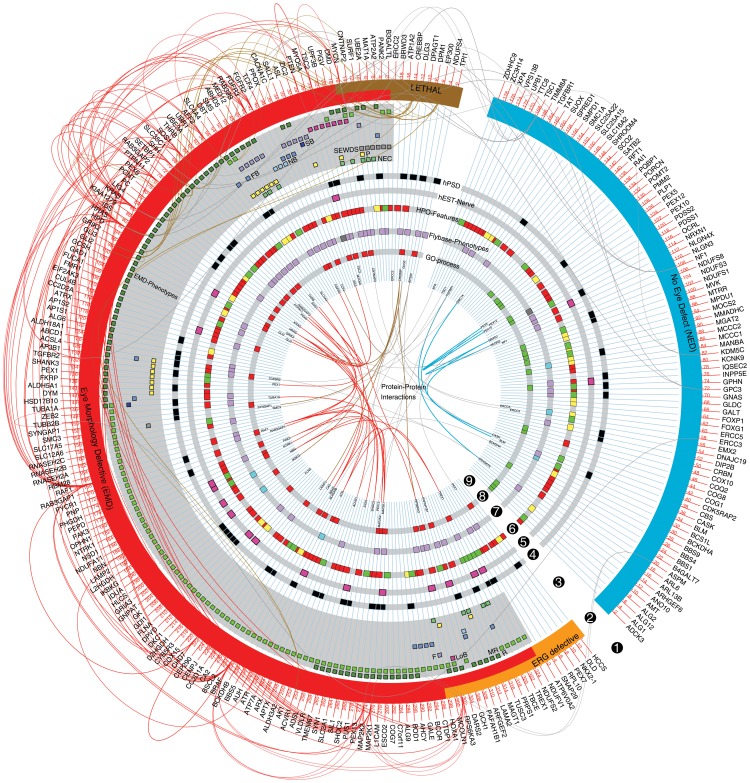
The modular landscape of Intellectual Disability. Graphic summary of ID genes, phenotypes and features identified in this study. Note the consistent asymmetry of features among EMD- versus NED-ID genes in these datasets. From the periphery to the centre: segment **1.** Human gene symbols and reported genetic interactions. **2.** Major phenotype classes: EMD (in red), ERG defective (in orange), NED (in blue) and lethal (in brown) phenotypes. **3.** EMD categories. Rough (R), mildly rough (MR), long bristles (LoB), (partially) fused ommatidia (F), stubble bristles (SB), fewer bristles (FB), no bristles (NB), small eye, wrinkled/dented surface (SEWDS), loss of pigmentation (P), necrosis (NEC). **4.** Black squares: human postsynaptic density proteins (listed in **Table S3**). **5.** Pink squares: genes with their highest relative expression in nerve tissue (see also **Figure S2A**). **6.** Human phenotype ontology features (from HPO database, see Materials and Methods). Red: enriched for Head-Neck/Musculoskeletal features, green: enriched for metabolism, yellow: enriched for both terms. **7.** Significantly enriched phenotypes from FlyBase. Purple color represent nervous system related phenotypic terms (neuroanatomy, neurophysiology and photoreceptor) whereas turquoise color represents stress response phenotypes. Dark grey: both enriched. **8.** ID genes that contribute to enriched neuronal functions among EMD-ID genes (in red) and enriched metabolic process among NED-ID genes (in green). See **Figure S2C,D** for a the underlying GO terms. **9.** Protein-protein interactions (PPI). PPIs within EMD-, NED-ID and lethal gene products are represented as red, blue and brown colored lines, respectively. Grey lines represent PPI links between EMD or lethal to NED gene products.

In summary, human orthologs of EMD-ID genes tend to be more specific for the nervous system than the NED-ID gene orthologs with respect to their expression at the RNA and protein levels and with respect to the pathways they are involved in. The above determined fly phenotypes, human gene expression and annotated functions were plotted in a circos diagram to provide a global view of ID gene properties and to illustrate the consistent asymmetry in this composite landscape of ID (**Figure 5**, segments 2–8; a zoomable electronic version of the circos is provided as **Figure S3**). Annotated genetic interactions (DroID) and protein-protein interactions (PPI; from HPRD) between ID genes were also retrieved and integrated (**Figure 5**, segments 1 and 9). Interestingly, ID gene-encoded proteins have more than three times as many PPIs with each other as random proteins (PIE = 3.1; p<0.0001; taking into account the systematic biases in PPI networks for intensely studied genes that are caused by their high number of measured interactions [26]). These data substantiate that ID genes operate in common pathways. Restricting the analysis to human EMD-ID gene orthologs increased this connectivity, not just relative to the PPI database (PIE = 5.8; p<0.0001), but also relative to all screened ID genes (PIE = 1.7; p = 0.003). NED-ID gene orthologs also showed increased connectivity (PIE = 8; p<0.0001) relative to random proteins from the PPI database. The different biology of EMD-ID versus NED-ID orthologs that we observed at the pathway level is therewith supported by an enrichment of protein interactions within each class. The finding that ID genes show a high connectivity is, given their heterogeneity, not trivial.

### Molecular Connectivity, Modules and Biological Coherence of ID Genes

To shed light on the functional connectivity of ID, we further examined *Drosophila* genetic interactions, comprehensive protein interaction data (HPRD and human interologs) and co-purified protein complexes (DPIM) and integrated these connections with the phenotypes we obtained. Strikingly, connections among mildly rough and among rough ID genes were each 6 fold enriched over randomly chosen *Drosophila* ID genes (p<0.0001). Connections between long bristles genes showed 20 fold (p<0.002), and connections between other bristles phenotype categories 24 fold (p<0.001) enrichment relative to randomly chosen *Drosophila* ID genes. This modularity extends beyond the eye morphological phenotypes. Lethal genes showed an 18 fold enrichment (p<0.001), and the most enriched phenotype class, the ERG defective genes, reached 47 fold enrichment in homotypic interactions (p<0.002) (i.e. interactions between genes that fall into the same *Drosophila* phenotype category). Connections within the categories fused ommatidia, necrosis, loss of pigmentation, and small eye, wrinkled or dented surface have not yet been reported in any of the utilized databases. The identified enrichments in known connectivity validate the approach to map molecular modules in ID through *Drosophila* phenotyping.

**Figure 6:**
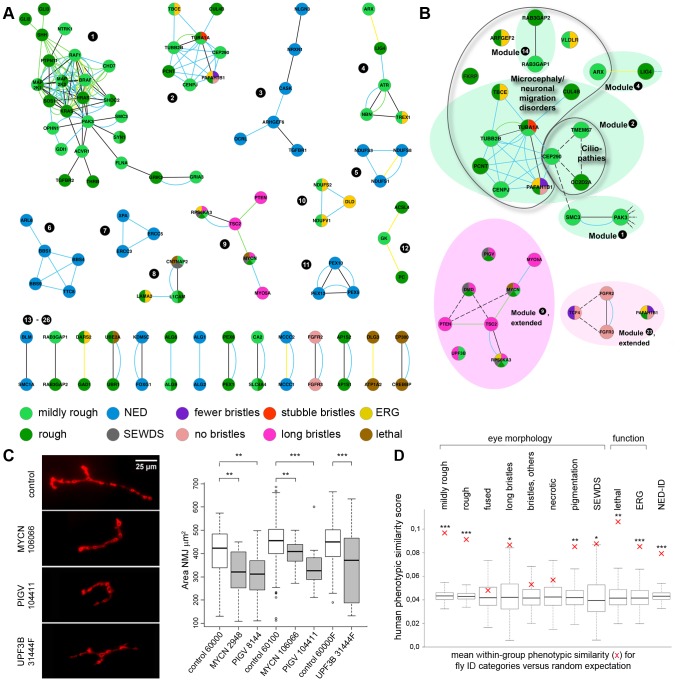
ID modules, proof of predictive value and phenotype coherence across evolution. (A) Phenotype-based homotypic ID modules. PPIs from HRPD in black, PPIs from human Interologs in turquoise, co-isolated protein complexes in yellow and genetic interactions in green. A high resolution image of Figure 6A is provided as **Figure S4.** (B) Three examples of homotypic modules that predict novel connections and phenotypes. Dotted lines indicate additional support identified by targeted literature search (see **Table S4**). (C) The ‘long bristles’ genes *MYCN*, *PIGV* and *UPF3B* are required, as predicted, for normal synapse development of the *Drosophila* larval Neuromuscular junction (NMJ). Anti-dlg1 labelling in red. The synaptic area (µm^2^) was quantitatively assessed using an in house-developed Fiji macro. Panels show representative NMJs. Box plots show the quantitative *MYCN*, *PIGV* and *UPF3B* synaptic phenotypes, compared to their appropriate genetic background controls. ** p<0.01; *** p<0.001; two tailed T-test. All phenotypes are highly significant. (D) Phenotypic similarity of human disorders caused by genes in the same fly eye phenotype category. Red crosses indicate the mean within-group phenotype similarity score. Box plots display the distributions of 1000 random controls sampled from the full set of genes in HPO, with the box representing the 25%–75% interquartile range. Asterisks indicate significant within-group phenotype similarity. ** p<0.05; ** p<0.01; *** p<0.001. Eye morphology categories as indicated, whereby “fused” represents fused and partially fused ommatidia, “bristles, others” represents fewer, no and stubble bristles, and SEWDS represents small eye and wrinkled or dented surface. Note that genes associated with ERG defects, lethal, and NED-ID genes (no eye morphology phenotype) also show a high degree of phenotypic coherence in human.

We next mapped the phenotype-based homotypic ID modules that are underlying the determined enrichments in connectivity among our phenotype categories (see Materials and Methods). In total, we identified 26 functionally coherent ID modules composed of 100 *Drosophila* ID genes and 200 homotypic connections (**Figure 6A** and its high resolution image provided as **Figure S4**). For the remaining 170 ID genes (63%), no homotypic connections were annotated.

### The *Drosophila* Long Bristles Phenogroup Successfully Predicts a Role for MYCN, PIGV and UPF3B in Synapse Development

Since *Drosophila* phenogroups showed high enrichments in known connectivity, they should be able to accurately predict novel gene functions and phenotypically relevant connections. To test this hypothesis, we further investigated the previously undocumented phenotype of abnormally long bristles, which identified a group of eight *Drosophila* ID genes. Five of these genes, *PTEN*, *TSC2*, *RPS6KA3*, *MYCN* and *Myo5A*, form a connected module (**Figure 6A,B**, module 9) associated with cancer biology [27]–[29]. In addition, *PTEN*, *TSC2*, *RPS6KA3* and *Myo5A* also play a role in synapse development and plasticity in post-mitotic neurons [4], [30]. Therefore our data suggested an unappreciated role for MYCN, the fifth protein in the module, in this process. To address this prediction, synapse development at the *Drosophila* larval Neuromuscular junction (NMJ) was quantified. The NMJ is a well-established model synapse that has already provided a number of fundamental insights into ID gene function and pathways [10], [24]. Pan-neuronal knockdown of MYCN in larvae caused abnormally small synapses (**Figure 6C**). We also predicted a role in synapse development for the remaining three long bristles genes *PIGV*, *UPF3B* and *DMD* (encoding dystrophin). Indeed, not only does loss of dystrophin affect synaptic transmission [31] and has recently been found to cause susceptibility to malignant tumors in mice [32], it also affects activity of Akt [33], a kinase that directly regulates TSC2. DMD may thus connect to the long bristles module and act upstream of Akt-TSC2 signalling in tumor and synapse biology. PIGV catalyzes a step in the GPI-anchor biosynthesis pathway, and UPF3B functions in nonsense-mediated mRNA decay (NMD). Both have not yet been implicated in synaptic development or cancer although other members of the PIG family and NMD factors have [34], [35]. Knockdown of PIGV and UPF3B, like knockdown of MYCN, caused a significant reduction in synaptic size (**Figure 6C**), consistently observed among RNAi lines. To address whether smaller synapses represent a phenotype that is common among *Drosophila* ID genes or whether these characterize the long bristles module more specifically, three further *Drosophila* ID gene sets of equal size were randomly selected from the modules and screened for synaptic growth defects. Of the three gene sets targeted by a total of 16 RNAi lines, only a single RNAi line caused a smaller synapse (6% vs. 100% of RNAi lines targeting long bristles genes; p<0.001, χ^2^) (**Figure S5**). A further single RNAi line in another gene set caused an increase in synaptic size (13% vs. 100% that cause any defect in synapse growth; p<0.01, χ^2^). No phenotypes were present in the third dataset, see **Figure S5**. Thus, *Drosophila* eye phenogroups can predict novel functions of *Drosophila* ID genes and connections between them. In addition to this experimental validation, a number of our predictions are further supported by targeted literature search (**Figure 6B** dashed lines, **Table 1** **,** **2** and **S4**, discussion). Further conclusions from our phenotype data and their suggested implications are indicated in **Table 1** and **2**. We conclude that our data add considerable information on ID gene functional connectivity, and provide a comprehensive, integrated picture of modular genotype-phenotype networks in our disease model.

**Table 1:** Predicted gene functions.

module	process	genes	genes predicted by phenotype to act in process	supported by data?	novel
**9**	synapse development/plasticity	MYO5A, TSC2, PTEN, RPS6KA3	MYCN, UPF3B, PIGV	this manuscript, **Figure 6C**	MYCN, UPF3B, PIGV
**1**	axon guidance	GLI2, GLI3, MAP2K1, MAP2K2, OPHN1, PTPN11, HRAS, SHH, SOS1, KRAS, RAF1	GDI1, CHD7, FLNA, GRIK2, KRAS, SYN1, ACVR1, PAK3, SHOC2, GRIA3, NTRK1, SMC3, TGFBR2, THRB	CHD7 [79], PAK3 [80], NTRK1 [81]	GDI1, FLNA, GRIK2, KRAS, SYN1, ACVR1, SHOC2, GRIA3, SMC3, TGFBR2 and THRB
**2**	mitotic cell cycle/mitosis	PCNT, CEP290, CENPJ, PAFAH1B1, TUBA1A, TUBB2B	CUL4B, TBCE	CUL4B [82]	TBCE
**2**	neuronal migration	TUBB2B, PAFAH1B1	PCNT, CEP290, CENPJ, TUBA1A, CUL4B, TBCE	PCNT [83], TUBA1A [84]	CEP290, CENPJ, CUL4B, TBCE
**3**	cell adhesion	NRXN1, CASK, NLGN3	ARHGEF6, OCRL, TGFBR1	ARHGEF6 [85]	OCRL, TGFBR1
**4**	DNA repair	LIG4, NBN, TREX1, ATR	ARX		ARX
**5**	nerve growth factor signalling	RPS6KA3, TSC2, PTEN	MYO5A, MYCN	MYCN [86], [87]	MYO5A
**23**	regulation of transcription	FGFR2, FGFR3, TCF4	PAFAH1B1		PAFAH1B1

Predicted gene functions. If several genes of a module have been implicated in a molecular process, other genes in the same homotypic ID module are predicted to act in the same process. Some of these predictions are already directly or indirectly supported by the indicated studies. Other predictions are novel, such as a role of MYCN, UPF3B and PIGV orthologs in synapse development. Numbering of phenotype modules as in **Figure 6A** **.**

**Table 2:** Predicted connections and wider implications.

module	gene(s)	predicted to connect to gene(s)	wider implications	supported by data?	novel
**9, extended based on phenotype (long bristle)**	Myo5A, MYCN, TSC2, PTEN, RPS6KA3	DMD, UPF3B and PIGV	1. DMD, UPF3B and PIGV act upstream in synapse and potentially in tumor biology. 2. DMD acts upstream of Akt-TSC2 signalling in synapse biology. 3. Cognitive defects in TSC2 and PTEN mouse models are reversible in adulthood. This phenotype module has implications for the prospects of therapeutic intervention with other module-associated disorders	this manuscript, **Figure 6C**; DMD signals to Akt [88]	UPF3B, PIGV
**2, extended (rough eye)**	CEP290	TMEM67, CC2D2A, SMC3	1.CEP290 connections with TMEM87 and CC2D2A link neuronal migration disorders to Ciliopathies. 2. CEP290 connection with SMC3 supports a recently proposed function of SMC3 in Planar Cell Polarity [89], a process crucial for Cilia [90] 3. Connects homotypic modules 1 & 2 via SMC.	TMEM67 [91], CC2D2A [92] and SMC3 [93]	
**2, extended (rough eye)**	TUBB2B, PAFAH1B1, PCNT, CENPJ, TUBA1A, CUL4B, TBCE, comprises microcephaly & neuronal migration disorders (lissencephaly and others)	RAB3GAP1, RAB3GAP2, ARFGEF2, FKRP, VLDLR; ARX, (microcephaly and lissencephaly)	1. Links microtubule-related neuronal migrations disorders to vesicle and protein trafficking. 2.Connects homotypic modules 2 & 4 via ARX		RAB3GAP1, RAB3GAP2, ARFGEF2, ARX, FKRP, VLDLR
**23, extended (no bristles)**	FGFR2, FGFR3	TCF4, PAFAH1B1	TCF4 regulates FGF signaling at the transcriptional level	TCF4, modENCODE [94]	PAFAH1B1

Beyond homotypic modules: selected connections predicated based on shared phenotypes and their wider implications. Further pair wise connections are listed in **Table S4**. Wider implications of these predictions are discussed in the results and discussion sections. Numbering of phenotype modules as in **Figure 6A** **.**

### *Drosophila* Phenotype Groups Show Phenotypic Similarity in Humans

Are the identified *Drosophila* phenotype groups relevant to humans? To test this, we asked whether the corresponding genes showed, in addition to ID, also other similar disease phenotypes. Using the Human Phenotype Ontology (HPO) database [36], we first determined that, relative to human orthologs of NED-ID genes, EMD-ID gene orthologs were enriched for morphological features of the head/neck (∼3 fold, 64 vs. 22 of top 200 features, p<10^−6^, χ^2^). In contrast, NED-ID gene phenotypes were enriched for disorders of metabolism and homeostasis (17 fold, 17 vs. 1 of top 200 features, p<10^−3^, χ2), which is consistent with the associated GO terms discussed above. We further inspected individual fly eye phenotype groups and determined their associated human mean phenotypic similarity scores [37]. This score reflects the degree of overlap between human disease features associated with each gene. To address the phenotypic similarity beyond ID, we excluded ID and all terms residing below it in the HPO hierarchy as features from the calculation of the similarity scores. Comparison of similarity scores in each phenotype group against the background expectation for all genes in the HPO database revealed that the phenotypic classes fused ommatidia, bristle phenotypes other than long bristles and necrosis phenotype classes showed no significant human phenotypic cohesion. In contrast, the remaining phenotype groups, mildly rough, rough, long bristles, loss of pigmentation, small eye and wrinkled or dented surface, lethal and ERG defective were each associated with significantly increased human phenotype similarity (**Figure 6D**). Moreover, NED-ID genes also showed highly significant coherence in their associated human phenotypes. This is consistent with their enrichment for disorders of metabolism/homeostasis and with the high connectivity among NED-ID genes, together validating them as an independent phenotype category and illustrating that in comparative functional studies also the absence of phenotypes can be informative.

Altogether, our findings demonstrate that *Drosophila* phenotype groups identify coherent disease phenotypes and highly connected functional modules among the large group of genetically heterogeneous ID disorders.

## Discussion

The number of genes that are known to cause Intellectual Disability is growing rapidly. Some phenotypic overlap can be observed among ID disorders and a number of ID genes have been proposed to operate in joint molecular pathways. Despite these interesting observations, to date neither a comparative phenotype annotation for ID genes nor a systematic integration of the genotype-phenotype network spaces [38] has been attempted. Here we have combined large-scale phenotyping and bioinformatics to systematically generate and analyze phenotypes that are associated with 270 human ID gene orthologs in *Drosophila*.

### Achievements and Limitations of the Chosen RNAi Approach

A previously validated transgenic RNAi library [12] was used as discovery toolbox in this study. Because our past work determined significant differences in knockdown levels induced by RNAi using this toolbox (20–60% of wt mRNA levels [39]–[42]) and because we consistently found morphological eye phenotypes with two independent RNAi constructs only for 54% of the investigated ID genes, it seems likely that a number of RNAi lines are not efficient enough to evoke phenotypes. To limit the impact of such false-negatives on our analyses, we included phenotypes caused by single RNAi lines. This strategy has been applied in previous RNAi screens using the same toolbox [14], [15], [43]. Although we cannot exclude the occurrence of false-positive and -negative findings on the single gene level, phototaxis and eye morphology proof of principle experiments were successful and reliably recapitulated previously reported mutant phenotypes (**Figures 1D** **and S1**). Twelve percent of *Drosophila* ID genes (33 genes) have annotated anatomical eye defects in Flybase. Most of these genes were reliably picked up in our screen (29 genes, 88%), indicating that the degree of false-negative hits is low (**Table S5**). High reproducibility of phenotypes was previously reported for RNAi lines with a high s19 specificity score of >0.85 [15]. In our screen, we were able to use lines with an s19 value of 0.98–1 in 97% of all cases (**Table S1B**), exceeding this standard.

There is evidence from the literature for (partial or complete) loss-of-function as the underlying disease mechanism in 93% of the ID genes/disorders investigated in our screen (see **Table S1A**). Therefore, knockdown by RNAi appears to represent a suitable approach to model most of the studied ID genes. For 6% of the investigated ID genes we found support for gain-of-function mechanisms. Most of these (affecting 9 of 15 genes) are activating mutations in the Ras-MAPK pathway. This may limit the conclusions that can be drawn for these genes from our phenotypes. Nonetheless, we note that loss of Ras-MAPK signalling also compromises cognitive functions in mouse and humans [4]. Our phenoclustering approach successfully grouped these nine Ras-MAPK components into a single phenotype module.

Close inspection of the determined homotypic modules (**Figure 6A**) showed that in few cases, genes that act in established common pathways or processes are divided over different modules due to their distinct *Drosophila* eye phenotypes. This is the case for NF1, a direct negative regulator of Ras proteins that does not group together with *HRAS* and *KRAS* genes (module 1), as well as for mitochondrial *NDUF* and peroxisomal *PEX* genes that are divided over different modules (5, 10 and 11, 20, respectively). Since the NED phenotype is involved, it is possible that some of these ‘splits’ are due to inefficiency of RNAi lines leading to false-negatives, as discussed above. However, others appear to reflect the biology of the genes/gene groups. For example, NF1, in contrast to the above discussed nine Ras-MAPK genes, is a negative regulator of Ras-MAPK signalling. It is therefore conceivable that its knockdown causes another phenotype (NED) than knockdown of the positive Ras-MAPK regulators (rough eye). A second negative regulator of this pathway, SPRED1, which has recently been found to directly interact with NF1 [44], is a NED gene as well. For the PEX genes, we would a priori have expected these to cluster together in our screen. It is worth noting though that the distribution of different PEX genes into phenotypic modules matches the molecular architecture of the peroxisomal machinery [45]. PEX1 and PEX6 (module 20) represent the two cytosolic AAA proteins that directly interact to form the peroxisomal export complex. In contrast, PEX10 and PEX12 (module 11) are both ring-finger proteins that directly interact with each other to form the ubiquitin ligase complex. This complex is required for matrix protein import and subsequent release of the cytosolic matrix protein receptor encoded by PEX5, the third PEX protein in module 11 [45]. In summary, the determined homotypic modules are unlikely to give an error-free and complete picture of biologically meaningful relations between the studied ID genes. However, the consistent properties of EMD- versus NED-ID genes, the high degree of known connectivity among our phenogroups, their increased phenotypic similarity in humans and the demonstrated validation of the predicted synapse phenotypes argue that false (negative and positive) discovery rates in this study are limited.

### Novel Functions of Genes Implicated in Intellectual Disability Disorders

In our screen, we identified more than 160 *Drosophila* ID genes that give rise to aberrant eye morphology, of which only 17% have been described previously on Flybase (**Table S5**). Furthermore, we identified 16 *Drosophila* ID genes that were required in the eye and in neurons for fly viability. Nearly half of these act in transcription or glycosylation-related processes. A further 21 *Drosophila* ID genes were required specifically for synaptic transmission or, more broadly, for basal neurotransmission. Histological analyses revealed that seven of these genes were essential for neuronal maintenance, whereas the majority was associated with functional defects despite structurally intact photoreceptors, implying that they impact neuronal transmission directly. CG7830, for example, is orthologous to two human non-syndromic ID genes, *TUSC3* and *MAGT1*. These two genes encode subunits of oligosaccharyltransferase complexes required for N-glycosylation [46], which have recently been found to possess Mg^2+^ transport activity [47]. In neurons, defects in TUSC3 and MAGT1-mediated Mg^2+^ homeostasis might thus directly impact Mg^2+^-dependent ion channels. All defects in basal neurotransmission that we identified in our study (**Figure 2B**) provide a cellular mechanism that can directly underlie cognitive deficits in patients.

### *Drosophila* as a Model for Human Phenomics of Genetically Highly Heterogeneous Disorders

Phenomics, the phenotype correlate of genomics, is an emerging discipline in biomedical research [38], [48], [49]. Despite recently established adequate data depositories such as the HPO database, human phenomics lags behind genomics [48], limiting the recognition of genetic networks based on human phenotype data. Furthermore, the often small number of patients per genetic condition and the impact of environmental factors limit progress in human phenomics and are likely to remain bottlenecks in disease research. Comparative phenomic analyses in model organisms can contribute to the identification of evolutionarily conserved genotype-phenotype correlations in the human disease landscape.

Which animal phenotypes are relevant to ID disorders? Apart from defects of the nervous system such as the synapse, learning and memory defects [50], [51], we here show that also less complex phenotypes can be informative. Phenologs are defined as phenotypes enriched among orthologous genes in two organisms [52]. They can be used to unbiasedly identify and predict human disease models, even when the relationship between the phenotypes is not immediately obvious. This is illustrated by the predictive value of a specific yeast growth phenotype as model for mouse angiogenesis defects [52]. In Flybase, the available information on eye phenotypes is limited. However, the total fraction of annotated morphological eye phenotypes is three times higher among *Drosophila* ID genes than genome-wide (12.2% of *Drosophila* ID genes with annotated eye defects (**Table S5**) vs. 3.9% genome-wide, p = 1.01e-09, hypergeometric test). Thus, eye phenotypes are more likely to associate with *Drosophila* ID genes than with random genes, suggesting that to a certain degree they can serve as phenologs of human cognitive dysfunction. Furthermore, genes associated in fly with the same phenotype group show significant phenotypic similarity also in humans, validating *Drosophila* as a model for human disease phenomics of genetically highly heterogeneous disorders.

### Functional Modules Underlying ID Disorders and Their Implications

Using the genotype-phenotype associations generated in this study, we found strong homotypic connectivity among ID genes. Integrating public interaction data with the generated *Drosophila* eye phenotypes led to novel insights in gene function and functional connectivity. In total, we detected more than two dozen homotypic modules. About half of these (14 of 26) are pairs. Thus, while informative, these clusters likely represent only a minority of all biologically relevant interactions. Some of the connections within modules are well established, such as the PPIs that delineate the Ras-MAP kinase signalling pathway at the core of the largest phenotype module (**Figure 6A**). Our phenotypes imply novel gene functions and functional connections within each of the established phenotype categories. The long bristles cluster successfully predicted that MYCN, PIGV and UPF3B are critical for synapse development. Other predictions remain to be tested experimentally, but a number of them are already supported by other studies (**Table 1** **,** **2** and **S4**). For example, despite lack of data in the utilized databases, the microtubule and neuronal migration-disorder related rough eye module two can be linked to other rough eye genes such as *CC2D2A*, *TMEM67* and *SMC3*, and potentially to other rough eye genes such as *Rab3GAP1*, *Rab3GAP2*, *ARFGEF2*, *FKRP*, *VLDLR* and *ARX* as supported by shared human neuronal migration phenotypes (**Figure 6B**, dotted lines). *CC2D2A*- and *TMEM67*-associated ID disorders are ciliopathies, and apart from its established role in chromosome cohesion, SMC3 has been recently shown to be required for Planar Cell Polarity, a process underlying cilium formation [53], [54]. These data therefore point to an intimate connection between neuronal migration disorders and ciliopathies. Indeed, a recent paper reported that migrating interneurons display dynamic primary cilia that carry receptors for guidance cues, the dynamics of which are disturbed in a ciliopathy [55].

Another example is the fused ommatidia phenotype (**Figure 3J′**), which resembles a phenotype previously reported in the literature as “glossy”. This phenotype has been proposed to identify genes with mitochondrial function [56], which is required for synaptic energy supply, receptor trafficking and calcium buffering. Indeed, among the twelve *Drosophila* ID genes in this phenotype category are the fly orthologs of *PPOX*, *SURF1* and *DBT*, three further genes with established mitochondrial function. Also *ASL*, a cytosolic enzyme of the urea cycle that partly takes place in mitochondria, gives rise to this phenotype. Four other fused ommatidia *Drosophila* ID genes encode regulators of transcription including *MED12*, a subunit of the mediator complex that in yeast has been shown to regulate transcription of genes with mitochondrial function [57]. In this context, it is important to note that functional connectivity between transcription factors and their target genes remains undetected in many databases, whereas this phenotype-based approach can identify or increase confidence in such relations. The “no bristles” category contains the *Drosophila* orthologs of *FGFR2*, *FGFR3*, *PAFAH1B1* (encoding Lis1) and the transcription factor *TCF4*, and comprises only a single annotated connection (*FGFR2*, *FGFR3*, **Figure 6A**). However, ModENCODE data show that the TCF4 ortholog *da* targets the two *Drosophila* FGF receptor genes *htl* and *btl* [58](**Figure 6B**), supporting further functional connections within this mini-cluster. Given the number of ID genes that encode transcription regulators, disruption of gene regulatory networks that comprise several ID genes are likely to contribute to the aetiology of ID.

### Translational Value of ID Modules

In the era of Next Generation Sequencing in human genomic research and diagnostics, the necessity to provide functional evidence of identified candidate disease genes is increasing exponentially. Here we have demonstrated that human disease phenomics in *Drosophila* is feasible, despite 1300 million years of evolutionary distance between the two species [59]. The identified genotype-phenotype modules, in combination with efficient fly phenotyping, should be applicable to facilitate identification of causative mutations among multiple DNA variants. Moreover, mapping molecular modules in ID provides a step towards network-based strategies that can target genetically heterogeneous patients with a common treatment. Recent research has demonstrated that cognitive defect in several animal models of ID are reversible in adulthood [60], [61]. Two of these genes, *PTEN* and *TSC2*, are part of the long bristles cluster, making other partners in this module attractive targets for genetic and pharmacologic rescue experiments and future clinical trials.

## Materials and Methods

### Human ID Genes and Orthology

ID genes were identified in the literature, in public and in-house databases, and manually curated by clinical specialists. Also conditions that might not be primarily regarded as ID syndromes (due to other prominent features or partial penetrance) were considered if independent genetic as well as independent clinical evidence for ID was found. Conditions with clinically or genetically low evidence or treatable metabolic conditions were not considered. To enrich for genes that act in neurodevelopmental processes underlying cognition, also genes associated with neurodegenerative manifestation (late onset), severe neurologic defects and early lethality were excluded. The orthologs of 390 ID genes (as of beginning of 2011) were determined using ENSEMBL's orthology classes (www.ensembl.org) and treefam annotations, including manual curation. One-to-one and one (fly)-to-many (human) orthologs were considered, identifying 285 fly orthologs. RNAi lines were available for 95% of these, which are subject of this study. In eight cases, two human paralogs are implicated in ID and have a common ancestor in *Drosophila*. *Drosophila* phenotypes and data associated with these were assigned to both human genes.

### Proposed Disease Mechanisms

Of the 270 investigated human ID disorders/genes, 200 are recessive (OMIM, the Online Mendelian Inheritance in Men database), and 28 further ID genes are reported to be haploinsufficient [62]. For 24 of the remaining 42 ID genes, evidence for (partial) loss-of-function as the underlying mechanisms exist (Pubmed, summarized on OMIM), illustrating that for >93% of ID disorders the pathomechanism is (partial) loss-of-function. In a very few cases (4/270) no data are available that would allow conclusions about loss versus gain-of-function as ID underlying mechanism. Support for gain-of-function mechanisms accounts for 5% (14/270) of the investigated ID genes.

### Fly Stocks and Breeding Conditions

Conditional knockdown of *Drosophila* ID genes was achieved with the UAS-GAL4 system [63], using a *w; GMR-Gal4; UAS-dicer2* driver [12], [19] and UAS-RNAi lines [12]. UAS-RNAi lines, their genetic background controls (60000, 60100) and *UAS-dicer2* (60009) were obtained from the Vienna *Drosophila* RNAi Centre (VDRC). *GMR-Gal4* (1104), *elav-Gal4; UAS-dicer2* (25750), *nonA^4b18^* (125), *norpA^45^* (9051), w^*^; sr^1^ ninaE^17^ e^s^ (5701) and w^*^; ort^1^ ninaE^1^ (1946) were obtained from the Bloomington *Drosophila* stock center (Indiana University). Crosses were cultured according to standard procedures and raised at 28°C unless indicated otherwise.

### Quality Control Criteria of RNAi Lines

Information collected in previous RNAi screens [14], [15], [43] was utilized to select genetic tools (GB and KK collections, see www.vdrc.at). ID lines from the site-integrated KK library were included in the primary screen. These lines bear no risk for gene disruption at the integration locus, ensure high expression and represent independent constructs that do not overlap with those of the GB collection. They are also characterized by minimized off-targets, reflected in high s19 values (**Table S1B**). Including the potent KK library in our screen allowed us to use lines with highly specific s19 scores of 0.98–1 in 97% of all cases.

### Phototaxis Assay and Index

A modified countercurrent apparatus was used to fractionate genotypes among six tubes, according to their visual activity (see **Figure S1**). The phototaxis index (PI) is calculated as ∑i*N_i_)/N, where N is the number of flies, i is the tube number, and N_i_ is the number of flies in the i^th^ tube. Average PI and standard deviation were calculated from three independent experiments on different test days. Assays were performed under standardized conditions, and progenies from control crosses served as internal controls. Populations of 40–70 flies, mixed sex, at the age of day 3–4 after eclosion and a walking time of 15 seconds were used. Based on the average PI of the control (PI = 5.2), and a maximal standard deviation of 1.2 per RNAi line, we defined a stringent cut-off of PI<4 to define a phototaxis hit.

### Scoring of Eye Morphology Defects

Eye morphology defects were scored by two independent experimentators. Despite a reported effect of *GMR-Gal4* driver constructs on eye development [64], our driver controls showed merely mildly rough phenotypes in a maximum of 10% of eyes. A mildly rough phenotype was therefore only scored if present in the majority (>90%) of knockdown eyes. No other eye phenotypes were observed in controls.

### Scanning Electron Microscopy (SEM)

Three to four days old females of the appropriate genotype were fixed in 1% glutaraldehyde, dehydrated by an ethanol series (25, 50 and 75%), critically-point dried and mounted on aluminum stubs. Samples were coated in gold by sputter coating and afterwards examined with a *JEOL 6310* SEM.

### Histology

Heads from 3–4 days old female progenies raised at 25°C were prefixed for 30 min in 2% glutaraldehyde buffered with 0.1 M Sodium cacodylate pH 7.4, bisected and fixed for another 24 hours. Bisected heads were postfixed for 1 hour in 1% Osmium teroxide in Paladebuffer pH 7.4 with 1% Kaliumhexacyanoferrat (III)-Trihydrat, dehydrated in ethanol and propyleenoxide and embedded in a single drop of Epon. Semi thin, 1 µm thick transverse and longitudinal sections were stained with 1% Toluidine Blue.

### ERGs

ERGs were performed as previously described [65]. Flies were tested at day one after eclosion. Per genotype eight to ten flies were recorded and the average of five representative recordings is shown.

### Quantitative Evaluation of *Drosophila* Synapse Development

Segment 2, 3 and 4 muscle 4 Type 1b neuromuscular junctions (NMJs) of wandering L3 panneuronal knockdown larvae were analyzed after dissection, a 30 min fixation in 3.7% PFA and immunolabelling with an anti-discs large 1 antibody (anti-dlg1, supernatant, 1∶25) (Developmental Studies Hybridoma Bank, University of Iowa). NMJ pictures were obtained using a Leica automated brightfield multi-color epifluorescence microscope. Images were automatically processed and the synapse area was measured by an advanced in house-developed Fiji/ImageJ macro. Mutant synapses were compared to their proper genetic background controls. For the X-linked UPF3B RNAi line 31444 and its control, exclusive female knockdown animals were selected. UPF3B RNAi line 31445 was not available at the stock centre for retesting. In contrast, for AP1S2, NDUFS8 and CHD7 independent RNAi lines were available at the time of synapse evaluation and have been utilized. At least 16 synapses were analyzed per genotype. Random sets of *Drosophila* ID genes subjected to NMJ analysis were determined from homotypic modules using a PHP script-based random number generator. Constraints were set on the min and max values and previously generated numbers were excluded to avoid duplicates. Independent sets of specified size were generated for subsequent analysis.

### Annotation of Fly Phenotypes

*Drosophila* ID genes were assigned to all phenotype categories that describe (an aspect of) the observed associated defects. Since RNAi induces variable knockdown that will in some cases not be sufficiently strong to evoke a loss-of-function phenotype, “single hit” genes were included in the further data analysis, as in previous *Drosophila* RNAi screens [14], [15], [43]. In any other scenario, one inefficient RNAi line would disqualify the efficient one, which would likely result in a large amount of false-negatives. For annotations of already known defects associated with EMD- and NED-ID or all *Drosophila* ID genes, the *Drosophila* genes annotated with defective phenotypic classes behavior, neuroanatomy, neurophysiology, behavior, photoreceptor, cell cycle and stress response phenotypes as well as with anatomy defective classes retina and photoreceptor cell were fetched from FlyBase (version march 2012) (www.flybase.org) [66]. A hypergeometric distribution test was carried out to check the enrichment of these phenotypes within EMD-ID and NED-ID genes against the background of (fly) phenotypes associated with all *Drosophila* genes that have orthologs in human.

### Assessing Tissue Expression

EST profiles from cDNA libraries of 45 normal human tissues were retrieved from the NCBI UniGene database [67] (ftp://ftp.ncbi.nih.gov/repository/UniGene/Homo_sapiens/Hs.profiles.gz) and expression abundance for each gene across the tissues was calculated. Since average expression between tissues varied significantly, we ranked genes in each tissue according to their expression levels. Subsequently we determined for each gene the tissue of its highest normalized expression as the one in which the gene had its highest rank.

### Gene Ontology Analysis

Overrepresentation of GO biological process and pathway terms for human EMD- and NED-ID gene orthologs against the human genome background data sets were identified using the Database for Annotation, Visualization and Integrated Discovery (DAVID) v6.7, web based program [68], [69].

### Interaction Network Datasets and Analyses

Direct physical protein-protein interaction data sets (HPRD_Release9_041310.tar.gz) from the Human Protein Reference Database (HPRD [70]) were downloaded and used as the standard protein interaction data for our study. Human interologs [71] (containing interactions from HPRD, BioGRID, IntAct, MINT, and Reactome; version 2012_04), DPIM-coAP complex data (protein interactions determined in large-scale co-affinity purification screens, *Drosophila* Protein Interaction Mapping project [72] (DPIM; version 2012_04), and *Drosophila* Genetic interaction data (version 2012_04) were downloaded from DroID (http://www.droidb.org/) [73], [74]. Physical interaction enrichment (PIE) scores of human orthologs of EMD- and NED-ID genes were calculated against HPRD, using the PIE algorithm with a minor modification in the normalization factor [26] to account for biases in the number of reported interactions for disease genes. Interaction enrichment scores for the specific phenotype categories within EMD, for lethal and for ERG ID gene products represent the number of unique connections determined from the combined interaction data sets per phenotype (HPRD, human interologs, DPIM-coAP complex and genetic interactions) divided by the number of connections for randomly (10,000 times) chosen ID genes from the combined interaction data sets.

### Circos Diagram

Circos-0.56, a freely available software package [75] was downloaded and used for the depiction of most phenotypes and significantly enriched features, determined as described above.

### Phenotype-Based Homotypic ID Modules and Visualization

The combined interaction data sets (see ‘Interaction network datasets and analyses’ above) were loaded into and visualized with the Cytoscape v2.8.1 tool [76]. Different phenotypes were colored using the MultiColored Nodes plug-in v2.4.0 [77]. Homotypic phenotype modules were identified among the entire ID interactome using Cytoscape's v2.8.1 ‘create new network from attribute’ algorithm. The phenotype-based homotypic ID modules are defined as connected genes with shared phenotype. Thus, genes with a non-overlapping phenotype cannot be part of the same phenotype-based module.

### Human Phenotypic Similarity

The Human Phenotype Ontology (HPO) [36] genes-to-phenotype mapping file, build 694, was downloaded from the HPO website (www.human-phenotype-ontology.org). This file maps genes to lists of standardized phenotypic features organized in a hierarchical structure (ontology). Phenotype similarity was determined based on these feature lists, using an adapted version [37] of a previously published algorithm [78] that takes the hierarchical structure into account. Basically, the human phenotypic similarity per gene pair was determined by calculating the correlation coefficient of the HPO feature vectors associated with each gene. The seven HPO features in the “Intellectual Disability” subtree were excluded from the feature vectors as the analyzed genes were selected based on this feature. Features were weighted according to their rarity and the number of features present in the vector. Before the feature vectors were compared, they were first supplemented with indirectly annotated features based on the feature hierarchy. This was accomplished by recursively adding parent features with progressively lower weights until the root of the feature hierarchy was reached. For each fly phenotype category, the mean pair-wise phenotypic similarity score was determined for all human genes associated with it. As a control, each set's score was compared with those of 1000 equal-sized sets of genes randomly sampled from the full list of HPO genes. For comparing the over-represented individual features of EMD-ID and NED-ID genes, we first identified the top 200 most significantly over-represented human phenotypic features for each gene set. This number was chosen to ensure that all considered features were over-represented at a corrected p-value threshold of 0.05 (Hypergeometric distribution; 206 and 563 features associated with NED-ID and EMD-ID genes respectively meet this threshold). Subsequently we determined what percentage of these specific features fall into the various top level HPO phenotypic categories, and compared these between EMD- and NED-ID genes.

## Supporting Information

Figure S1 Phototaxis procedure and proof of principle assays. (A)Schematic representation of the phototaxis device and assay, and formula to calculate the Phototaxis Index (PI). A fly population is placed into vial 1 and the vials are shifted (step I.). Flies are forced to the bottom of the vial (II.), the device is placed horizontally and flies are allowed to walk towards a light source into vial 1b for 15 seconds (III.). Vials are shifted (IV.) and flies that responded to light end up in the next bottom vial (V.). This procedure is repeated five times, which distributes flies according to their phototactic activity. (B) Proof of principle phototaxis assays with blind mutants. Genotypes and PI values are indicated. (C) Proof of principle phototaxis assays with *UAS-RNAi* lines corresponding to the tested blind mutants. Different conditions (*GMR-Gal4* and *GMR-Gal4; UAS-dicer2* drivers and breeding temperatures of 25 and 28°C) have been tested.
(TIF)

Figure S2 Enriched features of EMD- versus NED-ID genes. (A) EST expression profiling of human EMD- and NED-ID gene orthologs compared against the whole human genome in “nerve” tissue, the tissue with the largest fraction of EMD orthologs among all 45 tissues analyzed, and in four tissues that show representative profiles (**p<0.01, ***p<0.001). (B) Significantly enriched FlyBase phenotype terms associated with either EMD- or NED-ID genes, or both (*p<0.05, **p<0.01, ***p<0.001, Hypergeometric distribution test). (C,D) Functional enrichment of EMD- and NED-ID genes in GO-FAT biological processes and KEGG pathways (DAVID). All depicted terms are significantly enriched (***p<0.001) and have <1% false discovery rate. (A–D) EMD-ID: Eye morphology defective *Drosophila* ID genes; NED-ID: No eye phenotype *Drosophila* ID genes; Fly: all fly orthologs of human genes. HumanGenome: EST tissue expression of all the human genes in the UniGene database.
(EPS)

Figure S3 Zoomable Circos, electronic high resolution file of Figure 5.
(TIF)

Figure S4 Homotypic ID modules, electronic high resolution file of Figure 6A.
(EPS)

Figure S5 Quantitative synaptic area for three random sets of *Drosophila* ID genes. Box plots show the quantitative synaptic phenotypes for three gene sets of three *Drosophila* ID genes, randomly picked from the homotypic modules. Each of the 16 RNAi lines was compared to its appropriate genetic background controls. Synaptic area (µm^2^) was quantitatively measured by an in house-developed Fiji macro in an a procedure identical to measurements of MYCN, PIGV and UPF3B synapses. ** p<0.01; *** p<0.001; two tailed T-test.
(TIF)

Table S1 Data tables RNAi ID screen and results. (A) Human ID genes, proposed disease mechanism (see Materials and Methods), corresponding fly orthologs and transformant identities (order numbers) of the vdrc UAS-RNAi lines utilized per gene. (B) Main table listing identified phenotype information for all investigated RNAi lines, including phenotypes acquired in all performed primary and secondary assays as listed in Figure 1a (lethality, phototaxis, external morphology, ERG, histology upon GMR-mediated knockdown), and lethality upon panneuronal knockdown. (C) Phenotype groups. ID genes sorted by their phenotypes. Note that a gene is assigned to multiple phenotype groups when presenting with multiple phenotypes.
(XLS)

Table S2 Novelty of functional and histological data on 25 *Drosophila* ID genes with phototaxis defects. Table S2 indicates previous reports on the role of the identified *Drosophila* ID genes in phototaxis, ERG or other electrophysiology experiments, and related findings in mammalian systems. The novelty of eye morphology defects (FlyBase) is also indicated. Note that, to the best of our knowledge, most findings are novel.
(XLS)

Table S3 Identity of human EMD-ID and NED-ID gene orthologs among human Postsynaptic density proteins.
(DOC)

Table S4 Literature supporting the proposed novel functional connections between homotypic ID genes.
(DOC)

Table S5 *Drosophila* EMD-ID genes with known eye-related defects, extracted from Flybase.
(XLS)
