# Supplementary figures and images for: Human Intellectual Disability Genes Form Conserved Functional Modules in Drosophila
Source: PLoS Genet. 2013 Oct 31;9(10):e1003911. doi: 10.1371/journal.pgen.1003911 (PMC3814316; doi:10.1371/journal.pgen.1003911)

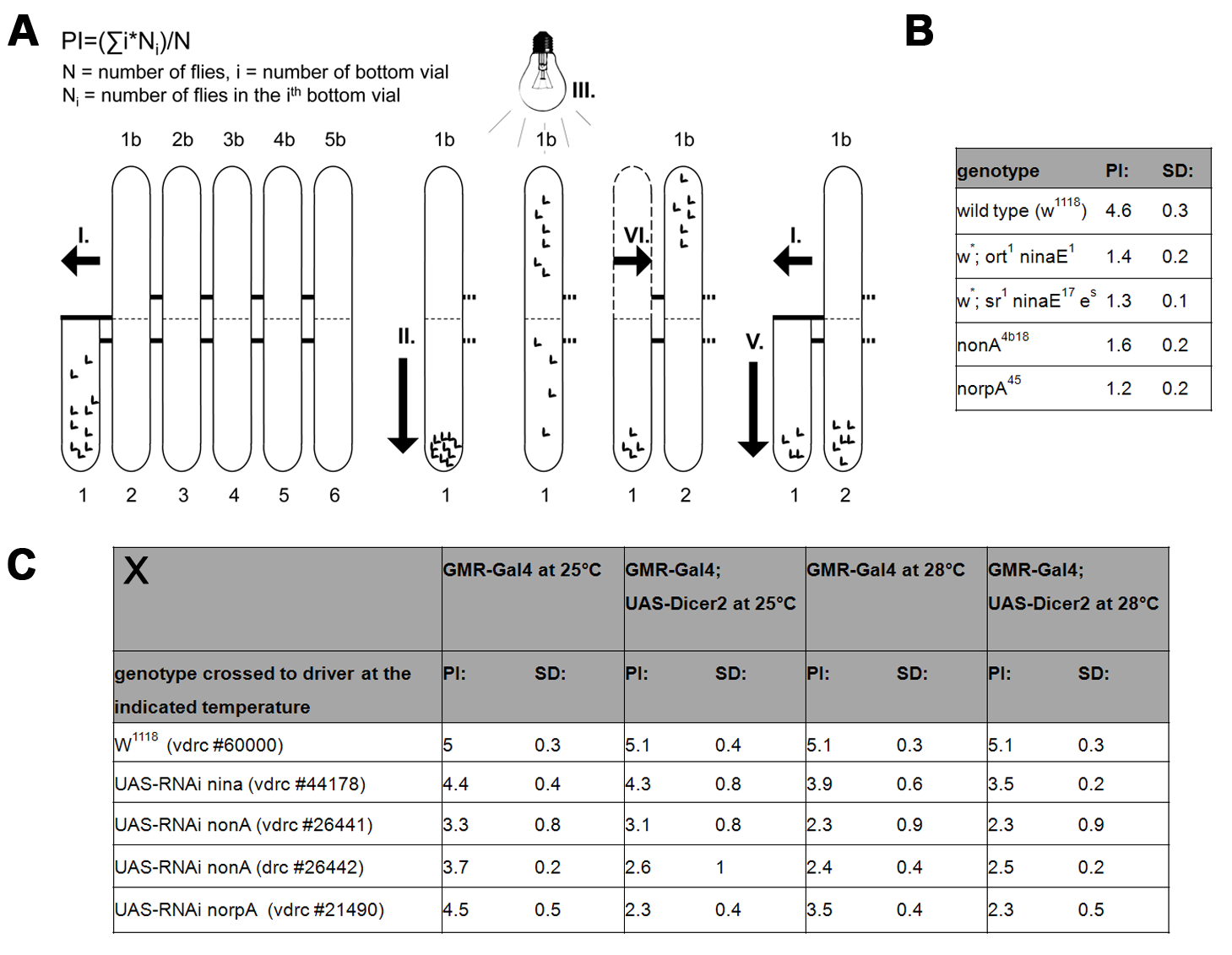

Supplement: Figure S1 — Phototaxis procedure and proof of principle assays. (A)Schematic representation of the phototaxis device and assay, and formula to calculate the Phototaxis Index (PI). A fly population is placed into vial 1 and the vials are shifted (step I.). Flies are forced to the bottom of the vial (II.), the device is placed horizontally and flies are allowed to walk towards a light source into vial 1b for 15 seconds (III.). Vials are shifted (IV.) and flies that responded to light end up in the next bottom vial (V.). This procedure is repeated five times, which distributes flies according to their phototactic activity. (B) Proof of principle phototaxis assays with blind mutants. Genotypes and PI values are indicated. (C) Proof of principle phototaxis assays with UAS-RNAi lines corresponding to the tested blind mutants. Different conditions (GMR-Gal4 and GMR-Gal4; UAS-dicer2 drivers and breeding temperatures of 25 and 28°C) have been tested. (TIF) [file pgen.1003911.s001.tif]

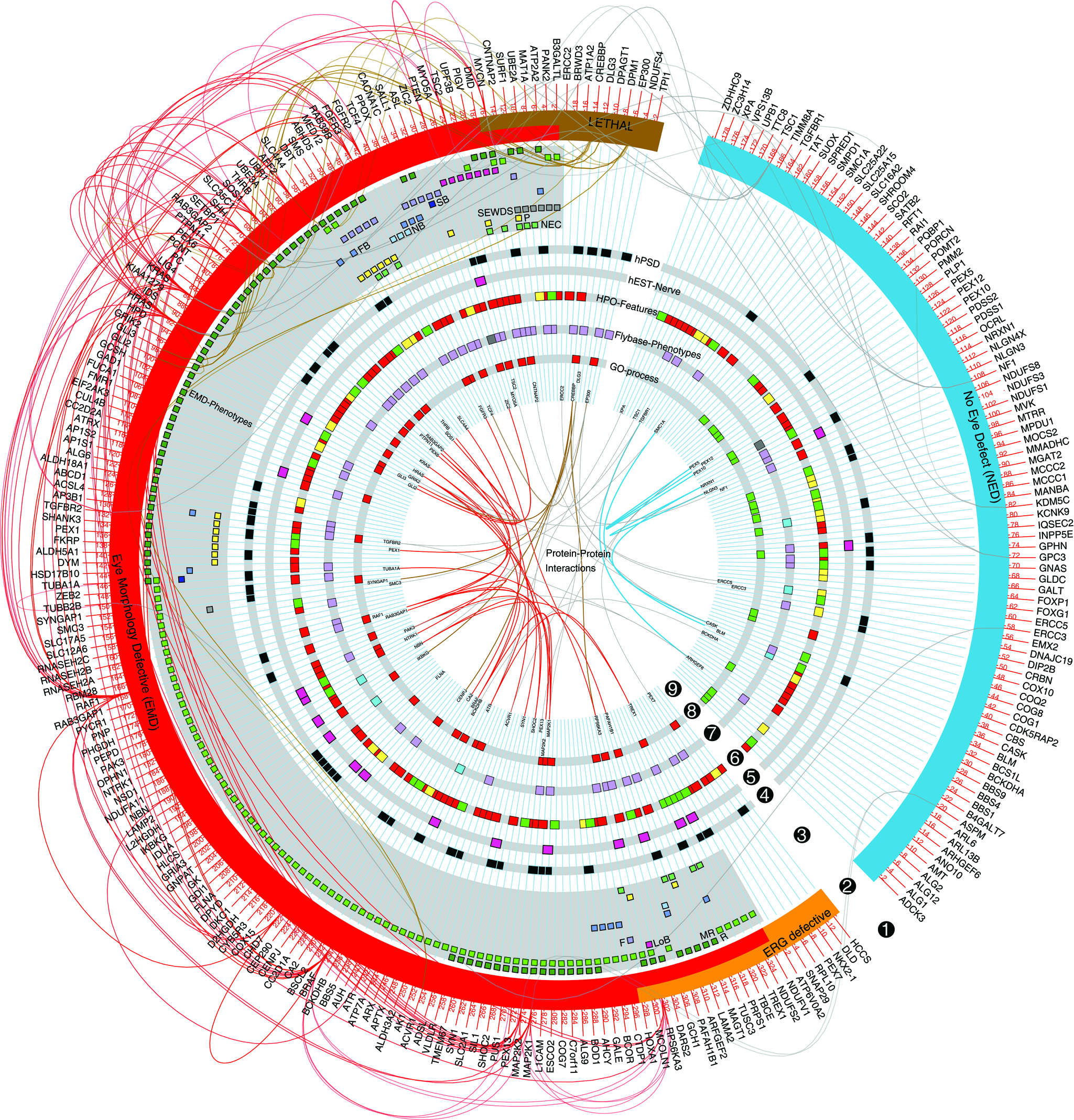

Supplement: Figure S3 — Zoomable Circos, electronic high resolution file of Figure 5. (TIF) [file pgen.1003911.s003.tif]

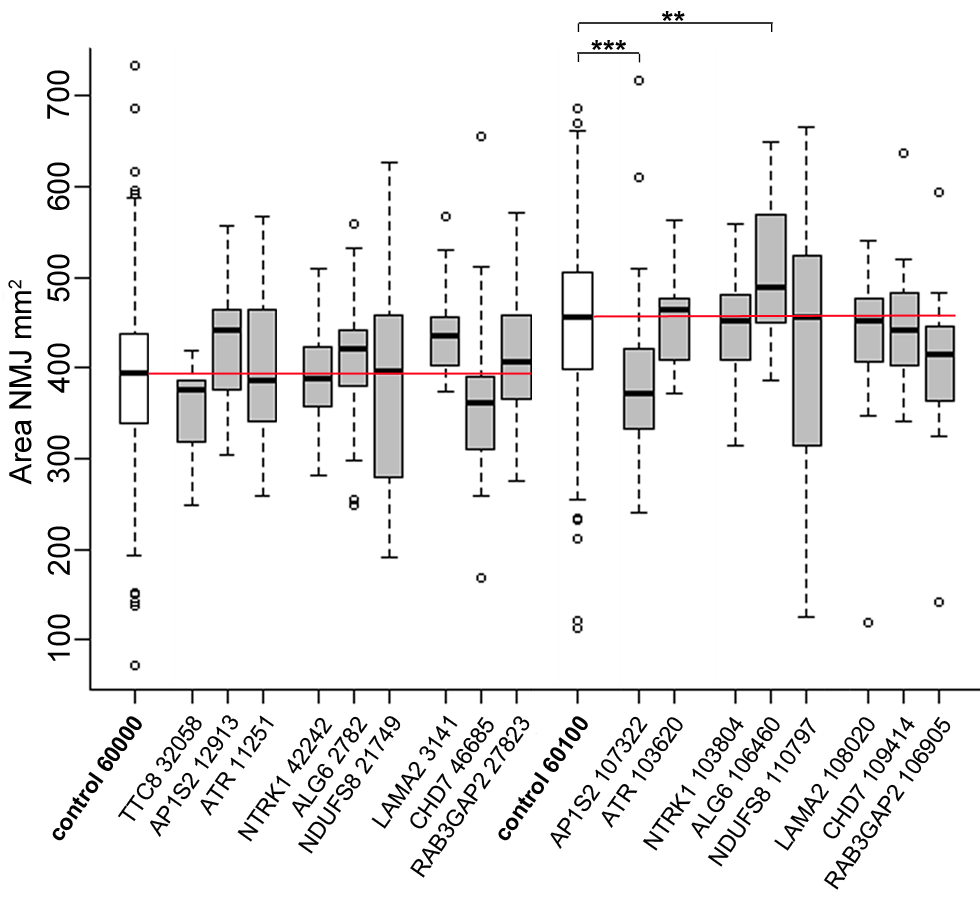

Supplement: Figure S5 — Quantitative synaptic area for three random sets of Drosophila ID genes. Box plots show the quantitative synaptic phenotypes for three gene sets of three Drosophila ID genes, randomly picked from the homotypic modules. Each of the 16 RNAi lines was compared to its appropriate genetic background controls. Synaptic area (µm2) was quantitatively measured by an in house-developed Fiji macro in an a procedure identical to measurements of MYCN, PIGV and UPF3B synapses. ** p<0.01; *** p<0.001; two tailed T-test. (TIF) [file pgen.1003911.s005.tif]
