# Supplementary material for: Human Intellectual Disability Genes Form Conserved Functional Modules in Drosophila
Source: PLoS Genet. 2013 Oct 31;9(10):e1003911. doi: 10.1371/journal.pgen.1003911 (PMC3814316; doi:10.1371/journal.pgen.1003911)
Supplement: Table S3 — Identity of human EMD-ID and NED-ID gene orthologs among human Postsynaptic density proteins. (DOC) [file pgen.1003911.s008.doc]

**Table S3. Identity of EMD-ID and NED-ID genes among human Postsynaptic density proteins.**

| **Entrez Gene ID** | **Gene Symbol** | **CG Symbol** | **FlyBase ID** | **EMD-ID or NED-ID** |
| --- | --- | --- | --- | --- |
| 215 | ABCD1 | CG2316 | FBgn0039890 | EMD-ID |
| 2182 | ACSL4 | CG8732 | FBgn0263120 | EMD-ID |
| 191 | AHCY | CG11654 | FBgn0014455 | EMD-ID |
| 203 | AK1 | CG17146 | FBgn0022709 | EMD-ID |
| 224 | ALDH3A2 | CG11140 | FBgn0010548 | EMD-ID |
| 7915 | ALDH5A1 | CG4685 | FBgn0039349 | EMD-ID |
| 1174 | AP1S1 | CG5864 | FBgn0039132 | EMD-ID |
| 8546 | AP3B1 | CG11427 | FBgn0003210 | EMD-ID |
| 10564 | ARFGEF2 | CG7578 | FBgn0028538 | EMD-ID |
| 488 | ATP2A2 | CG3725 | FBgn0263006 | EMD-ID |
| 549 | AUH | CG8778 | FBgn0033761 | EMD-ID |
| 760 | CA2 | CG7820 | FBgn0027844 | EMD-ID |
| 54862 | CC2D1A | CG4713 | FBgn0261983 | EMD-ID |
| 26047 | CNTNAP2 | CG6827 | FBgn0013997 | EMD-ID |
| 1629 | DBT | CG5599 | FBgn0030612 | EMD-ID |
| 2316 | FLNA | CG3937 | FBgn0014141 | EMD-ID |
| 2664 | GDI1 | CG4422 | FBgn0004868 | EMD-ID |
| 2710 | GK | CG18374 | FBgn0025592 | EMD-ID |
| 8443 | GNPAT | CG4625 | FBgn0040212 | EMD-ID |
| 2892 | GRIA3 | CG8442 | FBgn0004619 | EMD-ID |
| 3845 | KRAS | CG9375 | FBgn0003205 | EMD-ID |
| 3897 | L1CAM | CG1634 | FBgn0002968 | EMD-ID |
| 5604 | MAP2K1 | CG15793 | FBgn0010269 | EMD-ID |
| 5605 | MAP2K2 | CG15793 | FBgn0010269 | EMD-ID |
| 4644 | MYO5A | CG2146 | FBgn0261397 | EMD-ID |
| 4720 | NDUFS2 | CG1970 | FBgn0039909 | EMD-ID |
| 4723 | NDUFV1 | CG9140 | FBgn0031771 | EMD-ID |
| 5091 | PC | CG1516 | FBgn0027580 | EMD-ID |
| 26227 | PHGDH | CG6287 | FBgn0032350 | EMD-ID |
| 5631 | PRPS1 | CG6767 | FBgn0036030 | EMD-ID |
| 5781 | PTPN11 | CG3954 | FBgn0000382 | EMD-ID |
| 22930 | RAB3GAP1 | CG31935 | FBgn0051935 | EMD-ID |
| 25782 | RAB3GAP2 | CG7061 | FBgn0027505 | EMD-ID |
| 85358 | SHANK3 | CG30483 | FBgn0040752 | EMD-ID |
| 6513 | SLC2A1 | CG1086 | FBgn0261914 | EMD-ID |
| 8671 | SLC4A4 | CG4675 | FBgn0259111 | EMD-ID |
| 6853 | SYN1 | CG3985 | FBgn0004575 | EMD-ID |
| 8831 | SYNGAP1 | CG32560 | FBgn0261570 | EMD-ID |
| 7249 | TSC2 | CG6975 | FBgn0005198 | EMD-ID |
| 7846 | TUBA1A | CG1913 | FBgn0003884 | EMD-ID |
| 347733 | TUBB2B | CG9359 | FBgn0003889 | EMD-ID |
| 8573 | CASK | CG6703 | FBgn0013759 | NED-ID |
| 57609 | DIP2B | CG7020 | FBgn0024806 | NED-ID |
| 131118 | DNAJC19 | CG7394 | FBgn0036173 | NED-ID |
| 2778 | GNAS | CG2835 | FBgn0001123 | NED-ID |
| 10243 | GPHN | CG2945 | FBgn0000316 | NED-ID |
| 23096 | IQSEC2 | CG32434 | FBgn0026179 | NED-ID |
| 64087 | MCCC2 | CG3267 | FBgn0042083 | NED-ID |
| 4719 | NDUFS1 | CG2286 | FBgn0017566 | NED-ID |
| 4722 | NDUFS3 | CG12079 | FBgn0035404 | NED-ID |
| 9378 | NRXN1 | CG7050 | FBgn0038975 | NED-ID |
| 5354 | PLP1 | CG7540 | FBgn0037092 | NED-ID |
| 79751 | SLC25A22 | CG18347 | FBgn0260743 | NED-ID |
