# Supplementary material for: Human Intellectual Disability Genes Form Conserved Functional Modules in Drosophila
Source: PLoS Genet. 2013 Oct 31;9(10):e1003911. doi: 10.1371/journal.pgen.1003911 (PMC3814316; doi:10.1371/journal.pgen.1003911)
Supplement: Table S4 — Literature supporting the proposed novel functional connections between homotypic ID genes. (DOC) [file pgen.1003911.s009.doc]

**Table S4. Literature supporting the proposed novel functional connections between** homotypic ID genes

| **Gene 1** | **Module 1** | **Gene 2** | **Module 2** | **fly phenotype** | **reference ID** | **type of evidence** | **Synonyms** |
| --- | --- | --- | --- | --- | --- | --- | --- |
| KRAS | 1 | BRAF | 1 | rough | PMID:019661383 | functional connection |  |
| KRAS | 1 | SLC2A1 | Not in any module | rough | PMID:019661383 | functional connection | SLC2A1 = Glut1 |
| BRAF | 1 | SLC2A1 | Not in any module | rough | PMID:019661383 | functional connection | SLC2A1 = Glut1 |
| FLNA | 1 | KRAS | 1 | rough | PMID:017389601 | functional connection |  |
| FLNA | 1 | SOS1 | 1 | rough | PMID:012734206 | functional connection |  |
| GLI2 | 1 | GLI3 | 1 | rough | PMID:020512148 | functional connection |  |
| KRAS | 1 | GLI2 | 1 | rough | PMID:020512148 | functional connection |  |
| KRAS | 1 | GLI3 | 1 | rough | PMID:020512148 | functional connection |  |
| CEP290 | 2 | CC2D2A | Not in any module | rough | PMID:018950740 | PPI & genetic interaction |  |
| CEP290 | 2 | TMEM67 | Not in any module | rough | PMID:018327255 | genetic interaction | TMEM67 = MKS-3 |
| CEP290 | 2 | TMEM67 | Not in any module | rough | PMID:021725307 | complex association |  |
| CEP290 | 2 | CC2D2A | Not in any module | rough | PMID:021725307 | complex association |  |
| CEP290 | 2 | SMC3 | Not in any module | rough | PMID:16632484 | PPI |  |
| CASK | 3 | NF1 | Not in any module | NED-ID | PMID:011356864 | complex association |  |
| CASK | 3 | NF1 | Not in any module | NED-ID | PMID:020006588 | complex association | NF1 = neurofibromin 1 |
| PTEN | 5 | DMD | Not in any module | long bristles | PMID:019264909 | functional connection |  |
| PTEN | 5 | DMD | Not in any module | long bristles | PMID:020019182 | functional connection |  |
| MYCN | 5 | PTEN | 5 | long bristles | PMID:018278068 | functional connection |  |
| TSC2 | 5 | DMD | Not in any module | long bristles | PMID:016286242 | functional connection |  |
| GAD1 | 12 | ALDH5A1 | Not in any module | rough | PMID:007584821 | functional connection | ALDH5A1 = SSADH |
| GAD1 | 12 | GRIA3 | 1 | rough | PMID:019548263 | specific common phenotype in human |  |
| GAD1 | 12 | GRIK2 | 1 | rough | PMID:017553960 | functional connection |  |
| PEX6 | 19 | TGFBR2 | 1 | rough | PMID:014651998 | fictional connection & PPI |  |
| NDUFS2 | 20 | DBT | Not in any module | rough | PMID:019725078 | complex association |  |
| NDUFS2 | 20 | NDUFA11 | Not in any module | rough | PMID:01605643 | complex association |  |
| FGFR2 | 22 | TCF4 | Not in any module | no bristles | PMID:021177974 | functional connection |  |
| FGFR3 | 22 | TCF4 | Not in any module | no bristles | PMID:021177974 | functional connection |  |
| AP1S2 | 24 | RPS6KA3 | 5 | rough | PMID:020010893 | co-regulation |  |
| UBR1 | 25 | UBE3A | Not in any module | rough | PMID:016311597 | act in same process |  |
| UBE2A | 25 | UBE3A | Not in any module | rough | PMID:016909393 | act in same process |  |
| CC2D2A | Not in any module | TMEM67 | Not in any module | rough | PMID:021422230 | specific common phenotype in human |  |
| CC2D2A | Not in any module | TMEM67 | Not in any module | rough | PMID:021725307 | complex association |  |
